# Using Implementation Science to Examine the Impact of Cancer Survivorship Care Plans

**DOI:** 10.1200/JCO.2016.67.8060

**Published:** 2016-09-12

**Authors:** Rebecca Selove, Sarah A. Birken, Ted A. Skolarus, Erin E. Hahn, Anne Sales, Enola K. Proctor

**Affiliations:** Rebecca Selove, Tennessee State University, Nashville, TN; Sarah A. Birken, The University of North Carolina at Chapel Hill, Chapel Hill, NC; Ted A. Skolarus, University of Michigan; Veterans Affairs Health Services Research and Development Center for Clinical Management Research; and Veterans Affairs Ann Arbor Healthcare System, Ann Arbor, MI; Erin E. Hahn, Kaiser Permanente Southern California, Pasadena, CA; Anne Sales, University of Michigan, Ann Arbor, MI; and Enola K. Proctor, Washington University in St Louis, St Louis, MO

As strides have been made in cancer detection and treatment, the number of survivors of cancer in the United States has reached nearly 15 million. Survivors experience a number of significant gaps in post–cancer treatment care, including inadequate support for physical and emotional difficulties associated with cancer and its treatment, and poor communication and coordination among follow-up care providers, for example, oncologists and primary care providers.^1^ Survivorship care plans (SCPs) were recommended in 2006 by the Institute of Medicine (IOM) as a “common sense” approach to improving survivorship care.^1(p154)^

The IOM described SCPs as documents that include information regarding survivors’ cancer diagnosis and treatments, potential adverse consequences of those treatments over time, recommendations for screening for recurrence and prevention of other illnesses, information about employment and insurance access protection, and psychosocial service resources. SCPs also assign responsibility for managing ongoing survivorship care. SCPs are thought to be ideally delivered toward the end of cancer treatment—during patient-centered meetings with oncology clinicians—as tools to support patient engagement in discussions about prevention, follow-up care, and other concerns.^2^

Since 2006, several high-profile organizations, including the American College of Surgeons,^3^ the American Cancer Society,^4^ and others,^5-7^ have recommended or required the use of SCPs. These recommendations and requirements notwithstanding, evidence regarding the efficacy and effectiveness of SCPs is mixed at best. On the one hand, observational studies suggest that SCPs reduce the burden on survivors of synthesizing their records^8^ and function as a tool for improving communication and coordination of care among providers.^8-10^ On the other hand, five randomized clinical trials (RCTs) suggest that SCPs have little to no effect on desired outcomes.^11,12^

A decade after endorsement from the IOM and other organizations, SCP use remains limited. Birken et al^13,14^ found that more than one half of cancer programs (56%) reported they did not use SCPs at all, whereas Blanch-Hartigan et al^15^ found that < 5% of oncologists provided a written SCP to cancer survivors. Evidence also indicates that only one third of primary care providers always or almost always receive SCPs for survivors.^16^ Furthermore, these SCPs may not be comparable. Oancea et al^17^ explored the impact of treatment summaries and follow-up care instructions on depression among survivors and found that both SCP components were provided to only one third (32.5%) of survivors ≤ 5 years from diagnosis and to only one fourth (25.1%) of survivors who were > 5 years postdiagnosis. Parry et al^18^ have described multiple hurdles to widespread use of SCPs, including limited capacity to use technology for SCP development and ambiguity regarding responsibility for developing and implementing SCPs. Their recommendations for developing an evidence base for SCPs included expanding research beyond SCPs as documents to include the context and delivery process.

Limited SCP use may be framed, in part, as a consequence of challenges that are associated with their development and implementation in diverse contexts.^18,19^ These challenges may reflect differences of perceptions about what information, structures, and procedures are needed to improve communication among oncology and primary care providers and survivors to provide optimal care.^18,20^ In fact, readiness survey results and SCP implementation challenges led the American College of Surgeons Commission on Cancer to extend the phase-in period for its SCP Standard 3.3. Implementation challenges likely contribute to the conflicting evidence regarding the impact of SCPs^19^ and may help explain null findings.^21^ As such, SCP effectiveness may be difficult to determine without a well-defined intervention that is strategically implemented according to a plan that reflects key contextual elements. Currently, there is little guidance regarding best practices for implementing SCPs that take setting into account.^18^

Alfano et al^19^ described research tasks across phases of knowledge translation, from identification of promising therapeutic mechanisms to implementation of effective interventions in community practice. Although they presented the goals of each phase, including T2 (evaluation of interventions in phase III trials), T3 (providing understanding of barriers and facilitators of SCP interventions that were effective), and T4 (determining the impact of use of effective SCPs on population health), as sequential, they noted benefits of coordinating research across phases that “may be occurring simultaneously.”^19(p3)^ They also described as a barrier to advancement of survivorship care the dearth of research that addresses implementation of innovative interventions in community settings.

We appreciate the clarity and detail regarding each phase in the model presented by Alfano et al.^19^ We suggest an extension to their model with an approach that examines SCP effectiveness and implementation by combining tasks of phases T2 and T3 in the same research project. Fortunately, a research method for this alternative approach is part of the rapidly expanding field of implementation science.

In brief, implementation science—with strong support from the Division of Cancer Control and Population Sciences of the National Cancer Institute—is generally defined as the rigorous study of integration of evidence-based interventions into clinical and community health settings.^22^ Implementation science has evolved in response to concerns about the failure of many evidence-based interventions to improve health and quality of life, sometimes because they do not reach people who could benefit from them, and sometimes because they are not delivered as they were designed.^22^ The field provides frameworks^23,24^ and implementation strategies^25^ that can be used to address questions about contextual and process factors, including those that might influence SCP effectiveness. Implementation science methods could be applied to the process of developing evidence for SCPs, because contextual factors may be pivotal in determining whether delivery, content, context, or interactions among them, undermine their impact. Furthermore, combinations of implementation strategies^26,27^ may be needed to address setting-specific barriers to adoption of SCPs. Specifically, we propose that hybrid research designs^28^ be used to address questions about SCP effectiveness within an implementation science framework.

Implementation science frameworks have been used to improve survivorship care, for example, by guiding integration of evidence-based physical activity interventions^29^ and evaluating interventions to improve care for survivors of lung cancer.^30^ Use of implementation science to advance understanding and application of SCPs is consistent with a report by Parry et al,^18^ who advised attention to multiple levels of context as well as to the process of delivering SCPs. This also follows from the action plan developed by Alfano et al,^19^ which included “explicit attention to key tenets of implementation science.”^19(p8)^ Addressing implementation outcomes, such as acceptability and feasibility,^31^ that are associated with survivorship care interventions is critical because even minor inadequacies or errors in implementation may cause SCPs to fall short of their objectives,^32^ which leads to erroneous conclusions about their effectiveness.^33^

Landsverk et al^34^ provided an in-depth discussion of factors to be considered in selecting an implementation study design, noting that “there may remain questions about effectiveness of newly implemented strategies that are worth answering anew.”^34(p227)^ In this vein, we recommend the hybrid study design^28^ as a means for simultaneously assessing factors that influence SCP effectiveness with respect to clinical outcomes and factors that influence SCP implementation outcomes, such as adoption and sustainability.^31^

Curran et al^28^ described three types of hybrid designs, all of which combine methodologic elements that are generally associated with determining the effectiveness of an intervention, measured at the level of individuals, and strategies to address implementation barriers and facilitators at the delivery system level. In a type 1 hybrid study, the primary effectiveness outcome of interest is often treatment effect. For example, Highfield et al^35^ explored the impact of an evidence-based intervention to increase mammography among African American women and documented obstacles encountered during implementation. A type 2 hybrid research project attends equally to intervention effectiveness and implementation outcomes. This is exemplified in an evaluation of the process of implementing a care coordination program and also measuring tobacco cessation outcomes for patients of the Veterans Administration.^36^ A type 3 hybrid approach focuses primarily on implementation strategies and outcomes, such as acceptability and sustainability.^31^ This type of design was used to investigate the impact of different implementation strategies for a new model of integrated care.^37^ The care model sought to improve mental health outcomes, which were evaluated; however, the primary focus was on implementation outcomes that included fidelity to the intervention model and costs associated with implementation strategies.

Curran et al^28^ described type 3 designs as the approach of choice to evaluate the burden on a system for interventions driven by forces, such as patient demand or “respected consensus guidelines.”^28(p7)^ This type of hybrid design may be particularly well suited to address questions about SCPs, because we anticipate that to be effective, SCPs must be viewed as complex interventions “consisting of multiple behavioral, technological, and organizational components.”^38(p148)^ Like the interventions assessed in the hybrid studies previously described, SCPs seem to be reasonable solutions to a constellation of survivorship concerns. Research indicates that under some conditions, SCPs are beneficial and there is no evidence of harm.^29^

Central to SCP implementation are the perspectives of those who will use SCPs.^23,39^ Studies have described preferences of survivors of cancer and oncology, family, or general providers regarding SCP content, format, timing, and delivery.^9,40^ Survivors may be better served with SCP content and format that is tailored to their circumstances, for example, cancers and treatments,^41,42^ and characteristics, for example, age^41,43^ and ethnicity.^44,45^ Furthermore, oncology and primary care providers may require different kinds of information from an SCP.^46,47^ This is critical as the extent to which SCPs are acceptable to providers influences their willingness to use them.^13^ In addition, questions remain about optimal timing of SCP delivery relative to survivors’ diagnosis and treatment plan.^12,31^ Survivors whose treatments are relatively straightforward may benefit more from an SCP that is delivered directly after treatment. Survivors who are undergoing more complex treatment may not be ready for information an SCP provides until after a longer period has passed. In addition, some suggest SCPs may require boosters— updating SCPs and reviewing content with survivors at an optimally timed visit after the original SCP delivery visit—to be effective.^33^ Evidence also suggests that SCP effectiveness depends on delivery. Keesing et al^48^ found that most survivors preferred to have SCPs delivered by nurses, whereas some wanted to talk with a general practitioner or physician with an oncology focus about their survivorship care. A type 3 hybrid study could tailor SCPs to survivors’ preferences for SCP content, format, and timing of delivery, as well as assess patient–provider communication and patient engagement^49^ during the SCP meeting. Type 3 hybrid studies could test strategies that have been described by Powell et al,^26^ such as educating clinicians on the use of SCPs as a tool for promoting patient engagement and observing clinical outcomes such as stakeholder satisfaction. Another approach would be to study the content and delivery of SCPs in settings in which survivors are highly satisfied with their care coordination. Such studies would need to take into account and describe, with qualitative methods, the unique and complex characteristics of people and place that are relevant to outcomes.^50,51^

Our advocacy for the use of implementation science to study SCPs—a mandated clinical intervention for which evidence from RCTs is lacking—is congruent with calls for valuing a wider variety of research designs^52,53^ and expanding conceptualizations of evidence.^54^ Survivors and providers have first-hand experience with the processes and contexts in which SCPs are used. Their experience represents valuable evidence within implementation science investigations, and it is largely lost in existing RCTs that measure SCP effectiveness. Robust practice-based data collected in hybrid SCP studies could provide external validation of SCPs and the processes and contexts that accrue benefits to survivors and providers.^55^ More concisely, “if we want more evidence-based practice, we need more practice-based evidence.”^56^

In conclusion, it is clear that many survivors of cancer have significant unmet needs and that the clinical settings and communities in which they seek support and guidance are complex and varied. SCPs may be useful tools for addressing survivors’ problems; however, understanding what kind of SCP and delivery will be effective may be limited because of a lack of consideration of the influence of implementation on the effectiveness of SCPs. We contend that the effectiveness of SCPs is determined, in part, by context and delivery, and that advances in understanding the complex relationships among SCPs, survivors, providers, and setting would be facilitated by integration of implementation science methods, such as hybrid research designs.^28^
